# MR Micro-Neurography and a Segmentation Protocol Applied to Diabetic Neuropathy

**DOI:** 10.1155/2017/2761818

**Published:** 2017-04-16

**Authors:** P. F. Felisaz, G. Maugeri, V. Busi, R. Vitale, F. Balducci, S. Gitto, P. Leporati, A. Pichiecchio, M. Baldi, F. Calliada, L. Chiovato, S. Bastianello

**Affiliations:** ^1^Radiology Department, University of Pavia, Pavia, Italy; ^2^Postgraduation School in Radiodiagnostics, Università degli Studi di Milano, Milan, Italy; ^3^Unit of Internal Medicine and Endocrinology, IRCCS Salvatore Maugeri Foundation, Scientific Institute of Pavia, Pavia, Italy; ^4^Neuroradiology Department, C. Mondino National Neurological Institute, Pavia, Italy; ^5^Radiology Department, IRCCS Salvatore Maugeri Foundation, Scientific Institute of Pavia, Pavia, Italy; ^6^Institute of Radiology, IRCCS Fondazione Policlinico San Matteo, Pavia, Italy; ^7^Department of Brain and Behavioral Sciences, University of Pavia, Pavia, Italy

## Abstract

The aim of this study was to assess with MRI morphometric ultrastructural changes in nerves affected by diabetic peripheral neuropathy (DPN). We used an MR micro-neurography imaging protocol and a semiautomated technique of tissue segmentation to visualize and measure the volume of internal nerve components, such as the epineurium and nerve fascicles. The tibial nerves of 16 patients affected by DPN and of 15 healthy volunteers were imaged. Nerves volume (NV), fascicles volume (FV), fascicles to nerve ratio (FNR), and nerves cross-sectional areas (CSA) were obtained. In patients with DPN the NV was increased and the FNR was decreased, as a result of an increase of the epineurium (FNR in diabetic neuropathy 0,665; in controls 0,699, *p* = 0,040). CSA was increased in subjects with DPN (12,84 mm^2^ versus 10,22 mm^2^, *p* = 0,003). The FV was increased in patients with moderate to severe DPN. We have demonstrated structural changes occurring in nerves affected by DPN, which otherwise are assessable only with an invasive biopsy. MR micro-neurography appears to be suitable for the study of microscopic changes in tibial nerves of diabetic patients.

## 1. Introduction

Diabetic peripheral neuropathy (DPN) affects approximately 50% of patients with long-standing type 1 or type 2 diabetes. The onset of DPN is related to the duration of diabetes, the levels of hyperglycemia, and additional risk factors such as smoking, high body mass index (BMI), and hypertension [1]. Symmetric distal polyneuropathy is the most common form of diabetic neuropathy, typically occurring first in the lower extremities and then extending upward with proximal progression. Symptoms include motor impairment such as weakness and clumsiness of movement and sensory loss with a “glove and stocking” pattern of distribution. Tenderness, dysesthesia, numbness, tingling, burning, sharp pain, and neuropathic pain may also be present, but about 50% of the patients remain asymptomatic [2–4].

In common practice, the diagnosis of DPN is based on physical examination and electrophysiology. Physical examination should include simple standardized tests such as the Quantitative Sensory Testing (QST) that explores touch-pressure, vibration (by tuning fork), coolness and warmth, pain (pinprick test), and evaluation of the ankle jerk reflex. Electrophysiology studies are more sensitive and provide quantitative information, but they are limited by interrater variability and lack of standardized techniques [5]. More invasive tests include skin biopsy and sural nerve biopsy. Skin biopsy with intraepidermal nerve fiber density (IENFD) measurements may provide additional information at earlier stages, since the small sensory fibers are the first to be involved [6]. Both metabolic and ischemic factors are involved in the disruption of small fibers, which directly mediate the neuropathic pain. In advanced stages blood flow alterations and inflammation may lead to foot ulceration and amputation [7]. The sural nerve biopsy is less frequently required but may help in the differential diagnosis with other types of neuropathy [8]. Nerve biopsies in diabetic subjects show thickening of the base membrane, degeneration of pericytes, and endothelial cell hyperplasia, secondary to microvascular disease [9]. The inflammatory response is localized around the vessels of epineurium and perineurium [10]. In both symmetric and asymmetric neuropathy axonal degeneration is associated with nerve fascicles atrophy and fatty infiltration of the epineurium [11].

Magnetic resonance imaging (MRI) is a noninvasive technique with promising applications for the study of neuropathies. The several techniques already used in common practice, or still in their development stage, are known under the name of MR neurography. Typical protocols include high-resolution axial planes of the nerve trunks based on conventional turbo-spin echo sequences, T1 and fluid-sensitive weighting with fat suppression obtained with chemical shift selective pulses (CHESS), inversion recovery (STIR), or the Dixon method [12]. Interpretation of MR neurography is based on signal changes and nerve enlargement. Typically, the affected nerve displays an increased overall T2 signal, reflecting the presence of edema, hyperemia, or increased cellularity [13]. However, subtle changes are difficult to detect, and the lack of spatial resolution does not allow a clear-cut separation of the internal nerve components, which may be distinctly and differently damaged. Further issues are related to the variable orientation of the nerve along its course, which may lead to partial volume effects and magic angle artifacts, secondary to the anisotropic architecture of the nerve fibers [14].

MR microscopic techniques are less commonly reported but may play a role in this field. MR micro-neurography is a developing technique that pushes ahead the limits of spatial resolution, still using clinical high field scanners and surface coils. Within a smaller field of view it is possible to visualize anatomical details, such as the epineurium, the perineurium, and single fascicles, which otherwise would require a biopsy to be detected [15, 16].

There are detailed morphometric studies demonstrating increased fascicular area in nerves with DPN [17] as perineurial sheath expansion due to increased perineurial lamellar area and interlamellar space [18]. Previous studies have described as well endoneurial and epineurial alterations due to microvascular damage [19] and thickening of the epineurial sheath due to increased amount of connective tissue [20].

Since MR micro-neurography is capable of depicting some components of the internal nerve architecture, the aim of our study was to assess micro-structural changes in nerves affected by DPN. We have examined the nerve volume (NV), fascicle volume (FV), epineurial volume (EV), fascicles to nerve volume ratio (FNR), and nerve cross-sectional areas (CSA), comparing a population of subjects affected by DPN with a group of sex/age matched healthy volunteers. We have then assessed correlations of these parameters with the severity of neuropathy.

## 2. Materials and Methods

### 2.1. Patients

The procedures carried out in this study were in accordance with the ethical standards of the World Medical Association (Declaration of Helsinki). The ethical committee of our institution approved the study and examinations were performed after the acquisition of an informed consent from every subject. Inclusion criteria were as follows: age between 25 and 75 years, diagnosis of diabetes mellitus at least 5 years ago, and symmetric distal polyneuropathy confirmed by abnormal nerve conduction study. Exclusion criteria were as follows: unilateral neuropathy, compressive or posttraumatic radiculopathy, or the presence of other conditions that might be responsible for the neuropathy. Poor quality examinations were also excluded from the study after imaging acquisition (one subject was finally excluded due to the presence of obvious artifacts). The final study population included 16 patients (11 men and 5 women, age range 50–72, mean 60,9, and standard deviation 7,9). The severity of DPN was assessed using the revised Neuropathy Disability Score (NDS) [21] and two subgroups were created: mild neuropathy (NDS < 6) or moderate/severe neuropathy (NDS ≥ 6). We finally had 6 subjects with mild DPN and 10 with moderate/severe DPN. 15 healthy volunteers matched by age and sex (10 men and 5 women, age range 41–70, mean 54,9, and standard deviation 9,1) were evaluated by MRI as a control group.

### 2.2. Imaging Protocol

All images were acquired on a Discovery MR750 3 T scanner (GE Healthcare, Milwaukee, USA) [22] using a 6-channel carotid array coil, adapted for the study of the ankle region. The examinations were focused on the study of the tibial nerve at the medial aspect of the ankle, right above the tibial malleolus. Both sides were imaged in every subject. The protocol included a 3D SPGR sequence with standard fat suppression, short acquisition time (1-2 minutes), and anatomical coverage including the complete ankle region. Multiplanar reconstruction was used to select a perpendicular plane to the main axis of the tibial nerve and orient the next sequences. The second sequence was applied with a field of view located approximately 2 centimeters above the medial malleolus in a straight tract of the nerve, in order to obtain axial images to the nerve, minimizing partial volume effects. We used a higher resolution 3D SPGR with IDEAL (iterative decomposition of water and fat with echo asymmetry and least-squares estimation GE Healthcare, Milwaukee, USA) with an axial field of view of 5 × 5 cm and a longitudinal coverage of 2.8 cm. Details on the parameters that we used are listed in Table 1.

### 2.3. Postprocessing

One examiner performed the postprocessing blinded to the name of the subjects and their group (controls or neuropathic). The segmentation process used in the present study has been validated in a previous work [16]. The “in phase” and the “water” set of images were used from the four sets obtained with the IDEAL sequence (“water,” “fat,” “in phase,” and “out of phase”). The operator manually outlined the tibial nerve in every axial image (in total 14 slices) into all the “in phase” images, using the proprietary software JIM (Xinapse Systems Ltd, Essex, UK). On these images the external epineurial sheath was hypointense over a hyperintense background and served as a reliable segmentation border. This region of interest was then applied to the corresponding “water” image. In the “water” set of images, the fascicles and the perineurium appeared hyperintense and we assumed that the overall fascicle volume would be hyperintense over a dark background including the epineurial collagen and fat. Segmentation was performed with FAST (FMRIB Automated Segmentation Tool, Analysis Group, Oxford, UK) with a two-class algorithm based on signal and spatial intensity variation. This technique separated the hyperintense voxels (considered the fascicles) from the hypointense voxels (considered the epineurium). We obtained measurements of the fascicles volume (FV), epineurial volume (EV), and total nerve volume (NV). Then we calculated the fascicles to nerve ratio (FNR). We measured the nerve CSA with OsiriX (Pixmeo, Geneva, Switzerland), manually outlining the boundaries of the nerve and calculating the mean of three measurements at three random levels of the tibial nerve.

### 2.4. Statistical Analysis

Statistical analysis was performed with SPSS (IBM Corp., Armonk, NY, USA). Homogeneity of the groups was investigated using an independent sample *T*-test. The means of the NV, EV, FNR, and CSA were compared using an independent sample *T*-test. Differences between subgroups of neuropathy were tested using the two-tailed Mann–Whitney *U* test. Pearson correlation coefficient was used to assess relationships between NV, FV, FNR, CSA, and age.

## 3. Results

Subjects with DPN controls were found to be fairly similar for number (16 cases, 15 controls) and gender (11 men and 5 women in the DPN group, 10 men and 5 women in the healthy group). *T*-test for independent samples showed that age and sex distribution were not significantly different between the two groups.

**Figure 1:**
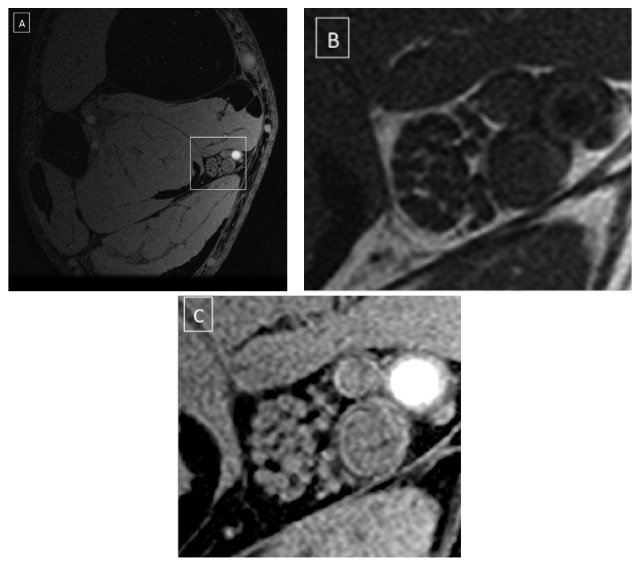
(A) Ankle, axial plane. Sequence: 3D SPGR IDEAL-water image. (B) Neurovascular bundle with the tibial nerve. Sequence: TSE T1 weighted. The nerve fascicles appear dark over a brighter background, corresponding to the epineurial fat. (C) Sequence: 3D SPGR-water image. The fascicles appear bright over a dark background corresponding to the epineurium. The perineurium can be seen surrounding some of the fascicles (see Figure 2).

**Figure 2:**
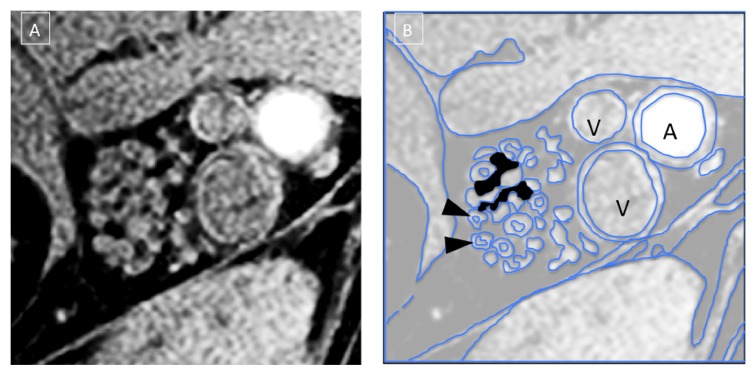
(A) Tibial nerve right above the level of the tibial malleolus, axial plane. Sequence: 3D SPGR IDEAL-water image. Voxel size is about 120 × 140 × 2000 *μ*m. (B) Diagram of the internal nerve aspect that is visualized. Arrowheads: two fascicles surrounded by the perineurium. Dark areas: epineurium. A: posterior tibial artery. V: posterior tibial veins.

**Figure 3:**
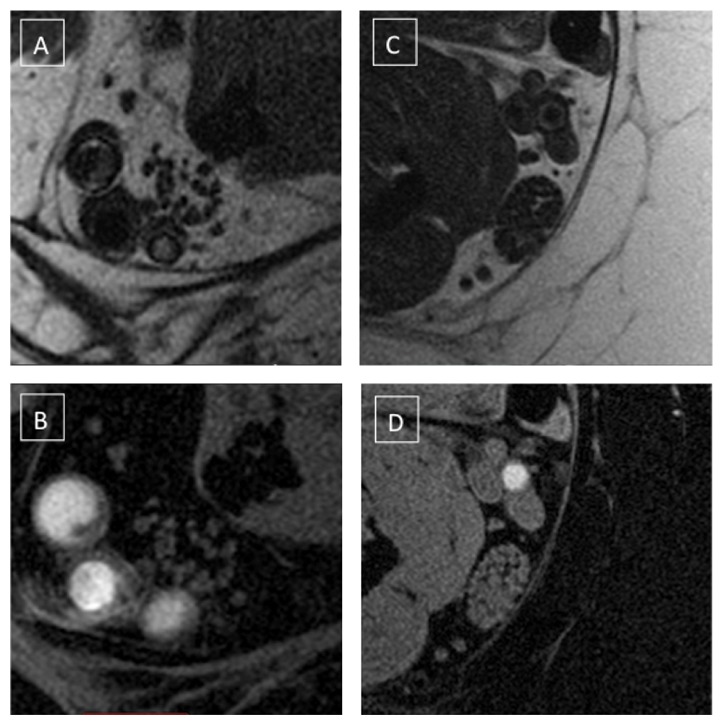
Tibial nerve with chronic degenerative changes (left (A, B)) compared with a volunteer (right (C, D)). The patient was in the group of the moderate/severe DPN and suffered from chronic pain and severe motor impairment. (A, C) Sequence: TSE T1. (B, D) Sequence: IDEAL-WATER. There is a visual increase of the interfascicular tissue (epineurium), while the fascicles appear reduced in number and area. Increase of fat and fibrous tissue within the epineurium is common in chronic stage of diabetic neuropathy.

The acquired images demonstrated the neurovascular bundle of the tibial nerve at the level of the ankle, right above the tibial malleolus. The IDEAL sequence provided a four-set images: “water,” “fat,” “in phase,” and “out of phase.” The “fat” images and the TSE T1 allowed a better depiction of the intraepineurial fat, with poorer contrast between the fascicles (Figure 1). The “water” images allowed the best characterization of internal nerve aspects: the fascicles appeared bright over a dark background corresponding to epineurium. The perineurium was a very thin hyperintense rim surrounding the fascicles and it was inconstantly visualized since affected by partial volume effects that limited the spatial resolution. Therefore perineurium segmentation was not reliable and this data was not collected (Figure 2). A visual increase of the epineurium as compared with the fascicles was seen in some DPN nerves, as shown in Figures 3(A)-3(B).

**Figure 4:**
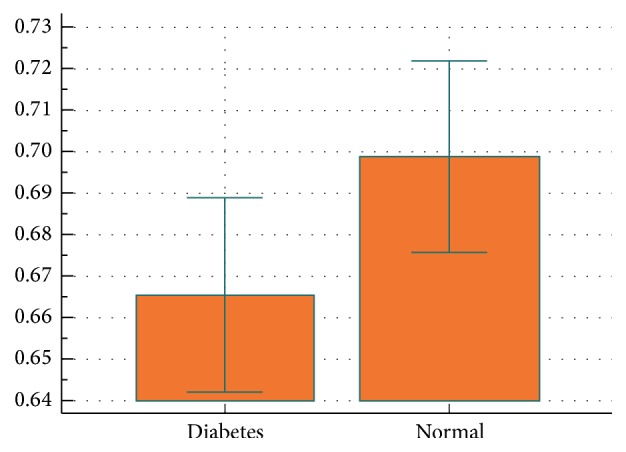
Data comparison graph for FNR in subjects with diabetic neuropathy (indicated with “diabetes”) and controls (indicated with “normal”). There is a significant difference between the two populations. For more details see Table 2.

The volumes measured with the segmentation technique are listed in Table 2. The total NV (considering 2.8 cm in the longitudinal axis of acquisition) was significantly higher in nerves with DPN than controls (361.9 versus 286.9 mm^3^, *p* = 0.028) (Figure 4, Table 2). FV was also increased in patients with DPN, but the difference did not reach statistical significance. On the other hand, the FNR was significantly lower in DPN than controls (DPN 0,665, controls 0,699; *p* = 0,040). CSA was significantly higher in DPN than in the healthy group (12,84 versus 10,22 mm^2^, *p* = 0,003).

Considering the subgroups on neuropathy, a statistical significant increase in NV and FV was only observed between the control group and the moderate/severe neuropathy. Similarly, the FNR was statistically decreased only between the control group and the moderate/severe neuropathy. CSA was significantly increased between the control and the mild neuropathy and between control and moderate/severe neuropathy, but not between mild and moderate/severe neuropathy (Table 3).

No statistical significant correlation was found between age, sex, and the parameters NV, FV, FNR, and CSA.

## 4. Discussion

The MR technique described in the present study allowed the investigation of some internal nerve components such as the FV, EV, and FNR that are not usually detected using conventional MR neurography. In subjects with diabetic neuropathy the NV and the CSA were increased while the FNR was decreased. CSA is a well-known parameter that may be increased in some neuropathies such as diabetic [23]. FNR instead is a novel parameter that indicates changes within the nerve (e.g., reduction in nerve fascicles), adjusted for the nerve volume and thus more independent from typical sources of intersubject variability such as body weight and height.

We observed also an increase of the FV, but statistical significance was only observed between controls and patients with moderate/severe neuropathy. This observation is in agreement with previous morphometric studies [17].

The decrease of the FNR in patients with DPN may be interpreted as a net increase of the epineurial fraction (EV). Also these results are in agreement with histopathology [20, 24]. Chronic damage secondary to long-standing diabetes results in fascicles atrophy and axonal degeneration, fibrous connective tissue proliferation, and fat accumulation. These nerve alterations eventually lead to a thickening of the epineurial layer.

The perineurium, a hyperintense rim surrounding the nerve fascicles, was inconstantly seen and was not considered as a parameter in the present study, although a thickening of the perineurial sheath would be expected in DPN.

The role of imaging in DPN is limited, but it might be useful when clinical examination or conduction studies are inconclusive. Diagnostic application fields might be the occurrence of one-side symptoms dominance, a pattern of distribution affecting a single nerve territory, or a rapidly progressive neuropathy. One of the main indications of MRI is the exclusion of a mass or a thickening of osteofibrous tunnels that may cause hindering and compression of the nerve. T2 signal is one parameter commonly used to assess nerve damage since increased intensity may reflect axonal degeneration and the presence of fluid within the fascicles and the interstitium [25]. T2 quantification has been used to identify nerve damage in DPN and to track it along the nerve course, showing alterations predominant at the proximal thighs rather than distal legs [26]. However, segmentation of the volume of fascicles and of epineurium may provide more detailed information, for example, which of the internal nerve compartment is enlarged, whether the epineurium is thickened, and whether the fascicles are swelling or atrophic.

Limitations of the present study may be as follows. FSL FAST is routinely used as a brain segmentation tool but it has seldom applications outside the brain; as for brain plaques quantification, the described nerve segmentation technique shares similar limitations, such as spatial resolution, contrast resolution, and signal to noise ratio. Improvement of MRI technologies is likely to lead to better image quality with easier distinction of the internal nerve components. Second, we have analyzed the tibial nerve and not the sural nerve, although only the latter has been commonly subject to biopsy. We used the tibial nerve since it is easier to study with our technique. Compared to the sural nerve, the tibial nerve has a greater fascicular area (2 to 4 times), increasing the signal to noise ratio and providing best results of segmentation.

This study focused on the MRI signs of chronic neuropathy. However the development of techniques that might recognize early neuropathic changes in diabetic patients is crucial, because it might impact the course of the disease. First, nondiabetes-related neuropathies may be discovered, some of which can be treated. In addition, there is a variety of treatment options for symptomatic DPN that, even though not altering the underlying disease or the natural history of diabetes, may positively impact the quality of life of these patients.

In conclusion, MR micro-neurography was able to show chronic changes occurring in nerves affected by DPN. In the future, the development of more accurate MRI protocols might replace invasive biopsies, allowing early detection of the disease and supporting the follow-up and the response to therapy.

## Figures and Tables

**Table 1:** Parameters of the MR sequences.

	Localizer	Fluid sensitive HR	Fat sensitive HR	T1-weighted HR
Type	3D SPGR	3D SPGR IDEAL	3D SPGR IDEAL	2D FSE
TR (ms)	5,9	16,9	16,9	650
TE (ms)	Min	Min	Min	Min
Flip angle	10	10	10	90
FOV (cm)	8	6	6	6
Acquisition matrix	128 × 128	512 × 420	512 × 420	512 × 416
Number of slices	128	20	20	11
Slice thickness (mm)	2	2	2	2
Gap (mm)	0	0	0	1
ETL	—	—	—	5
NEX	1	1	1	3
BW (KHz)	15	35	35	31
Fat sup.	Yes	No	No	No

**Table 2:** Comparison of parameters between subjects with diabetic neuropathy and controls. Data is expressed in mean ± SEM. Bold characters are used to enhance statistical significance. DPN = diabetic peripheral neuropathy. NV = mean of the nerve volumes (for a length of 28 mm). FV = mean of the fascicles volumes (for a length of 28 mm). FNR = fascicles to nerve ratio. CSA = mean of the cross-sectional areas. There is statistical difference (*p* < 0,05) in NV, FNR, and CSA between subjects with diabetic neuropathy and controls.

	DPN	Controls	*p*	Confidence interval (95%)	
				Inferior	Superior
NV (mm^3^)	361.85 ± 26.3	286.87 ± 18.0	**0.028**	−141.1	−8.87
FV (mm^3^)	239.12 ± 16.8	198.4 ± 12.8	0.065	−84.21	2.78
FNR	0.666 ± 0.011	0.699 ± 0.011	**0.040**	0.002	0.064
CSA (mm^2^)	12.84 ± 0.72	10.22 ± 0.45	**0.003**	−4.374	−0.854

**Table 3:** Test between the control group and the two subgroups of diabetic neuropathy. All data are expressed in mean ± SEM. All patients included in the diabetic neuropathy group had abnormal nerve conduction studies. Subjects with a neuropathy disability score of less than 6 points were considered “mild neuropathy,” and subjects with 6 points or more were considered “moderate/severe neuropathy.” Bold characters are used to enhance statistical significance. N.S. = Nonsignificant.

	Nondiabetic	Mild neuropathy	Moderate/severe	A versus B	B versus C	A versus C
	(A)	(B)	(C)			
NV	286.8 ± 18.0	326.7 ± 48.4	383.0 ± 30.6	N.S.	N.S.	**<0.01**
FV	198.4 ± 12.8	218.7 ± 29.7	251.4 ± 20.3	N.S.	N.S.	**<0.03**
FNR	0.699 ± 0.011	0.677 ± 0.018	0.659 ± 0.014	N.S.	N.S.	**<0.03**
CSA	10.22 ± 0.45	12.62 ± 1.27	12.97 ± 0.91	**<0.04**	N.S.	**<0.01**

## Abbreviations

BMI: Body mass index
QST: Quantitative Sensory Testing
IENFD: Intraepidermal nerve fiber density
MRI: Magnetic resonance imaging
EV: Epineurial volume
FV: Fascicles volume
NV: Nerve volume
FNR: Fascicles to nerve volumes ratio
CSA: Cross-sectional area
SPGR: Spoiled gradient echo
IDEAL: Iterative decomposition of water and fat with echo asymmetry and least-squares estimation
FAST: FMRIB Automated Segmentation Tool.

## References

[1] Adeghate E., Schattner P., Dunn E. (2006). An update on the etiology and epidemiology of diabetes mellitus. *Annals of the New York Academy of Sciences*.

[2] Davies M., Brophy S., Williams R., Taylor A. (2006). The prevalence, severity, and impact of painful diabetic peripheral neuropathy in type 2 diabetes. *Diabetes Care*.

[3] Pasnoor M., Dimachkie M. M., Kluding P., Barohn R. J. (2013). Diabetic neuropathy part 1: overview and symmetric phenotypes. *Neurologic Clinics*.

[4] Tesfaye S. (2011). Recent advances in the management of diabetic distal symmetrical polyneuropathy. *Journal of Diabetes Investigation*.

[5] Dyck P. J., Albers J. W., Wolfe J. (2013). A trial of proficiency of nerve conduction: greater standardization still needed. *Muscle and Nerve*.

[6] Shy M. E., Frohman E. M., So Y. T. (2003). Quantitative sensory testing: report of the therapeutics and technology assessment subcommittee of the American academy of neurology. *Neurology*.

[7] Johnson P. C., Doll S. C., Cromey D. W. (1986). Pathogenesis of diabetic neuropathy. *Annals of Neurology*.

[8] Sumner C. J., Sheth S., Griffin J. W., Cornblath D. R., Polydefkis M. (2003). The spectrum of neuropathy in diabetes and impaired glucose tolerance. *Neurology*.

[9] Behse F., Buchthal F., Carlsen F. (1977). Nerve biopsy and conduction studies in diabetic neuropathy. *Journal of Neurology, Neurosurgery and Psychiatry*.

[10] Younger D. S. (2010). Diabetic neuropathy: a clinical and neuropathological study of 107 patients. *Neurology Research International*.

[11] Said G. (2007). Diabetic neuropathy—a review. *Nature Clinical Practice Neurology*.

[12] Dixon W. T. (1984). Simple proton spectroscopic imaging. *Radiology*.

[13] Chhabra A. (2013). Magnetic resonance neurography-simple guide to performance and interpretation. *Seminars in Roentgenology*.

[14] Chappell K. E., Robson M. D., Stonebridge-Foster A. (2004). Magic angle effects in MR neurography. *American Journal of Neuroradiology*.

[15] Felisaz P. F., Chang E. Y., Carne I. (2014). In vivo MR microneurography of the tibial and common peroneal nerves. *Radiology Research and Practice*.

[16] Felisaz P. F., Balducci F., Gitto S. (2016). Nerve fascicles and epineurium volume segmentation of peripheral nerve using magnetic resonance micro-neurography. *Academic Radiology*.

[17] Malik R. A., Newrick P. G., Sharma A. K. (1989). Microangiopathy in human diabetic neuropathy: relationship between capillary abnormalities and the severity of neuropathy. *Diabetologia*.

[18] Ghani M., Malik R. A., Walker D. (1999). Perineurial abnormalities in the spontaneously diabetic dog. *Acta Neuropathologica*.

[19] Malik R. A., Tesfaye S., Thompson S. D. (1993). Endoneurial localisation of microvascular damage in human diabetic neuropathy. *Diabetologia*.

[20] Kundalić B., Ugrenović S., Jovanović I. (2014). Morphometric analysis of connective tissue sheaths of sural nerve in diabetic and nondiabetic patients. *BioMed Research International*.

[21] Young M. J., Boulton A. J. M., Macleod A. F., Williams D. R. R., Sonksen P. H. (1993). A multicentre study of the prevalence of diabetic peripheral neuropathy in the United Kingdom hospital clinic population. *Diabetologia*.

[22] Reeder S. B., McKenzie C. A., Pineda A. R. (2007). Water-fat separation with IDEAL gradient-echo imaging. *Journal of Magnetic Resonance Imaging*.

[23] Breiner A., Qrimli M., Ebadi H. (2016). Peripheral nerve high-resolution ultrasound in diabetes. *Muscle & Nerve*.

[24] Malik R. A. (1997). The pathology of human diabetic neuropathy. *Diabetes*.

[25] Thakkar R. S., Del Grande F., Thawait G. K., Andreisek G., Carrino J. A., Chhabra A. (2012). Spectrum of high-resolution MRI findings in diabetic neuropathy. *American Journal of Roentgenology*.

[26] Bäumer P., Weiler M., Bendszus M., Pham M. (2015). Somatotopic fascicular organization of the human sciatic nerve demonstrated by MR neurography. *Neurology*.

